# The treatment of advanced renal cell cancer with high-dose oral thalidomide

**DOI:** 10.1054/bjoc.2001.2025

**Published:** 2001-09

**Authors:** J Stebbing, C Benson, T Eisen, L Pyle, K Smalley, H Bridle, I Mak, F Sapunar, R Ahern, M E Gore

**Affiliations:** Department of Medical Oncology, The Royal Marsden Hospital, London, SW3 6JJ; HEAL Research Department, North Middlesex Hospital, London, N18 1QX; Chelsea and Westminster Hospital, Department of Clinical Neurophysiology, London, SW10 9NH, UK

**Keywords:** thalidomide, renal cell carcinoma

## Abstract

Thalidomide is reported to suppress levels of several cytokines, angiogenic and growth factors including TNF-α, basic fibroblast growth factor (bFGF), vascular endothelial growth factor (VEGF) and interleukin-6 (IL-6). The resulting anti-angiogenic, immunomodulatory and growth suppressive effects form the rationale for investigating thalidomide in the treatment of malignancies. We have evaluated the use of high-dose oral thalidomide (600 mg daily) in patients with renal carcinoma. 25 patients (all men; median age, 51 years; range 34–76 years) with advanced measurable renal carcinoma, who had either progressed on or were not suitable for immunotherapy, received thalidomide in an escalating schedule up to a maximum dose of 600 mg daily. Treatment continued until disease progression or unacceptable toxicity were encountered. 22 patients were assessable for response. 2 patients showed partial responses (9%; 95% CI: 1–29), 7 (32%; 95% CI: 14–55) had stable disease for more than 6 months and a further 5 (23%; 95% CI: 8–45) had stable disease for between 3 and 6 months. We also measured levels of TNF-α, bFGF, VEGF, IL-6 and IL-12 before and during treatment. In patients with SD ≥ 3 months or an objective response, a statistically significant decrease in serum TNF-α levels was demonstrated (*P* = 0.05). The commonest toxicities were lethargy (≥ grade II, 10 patients), constipation (≥ grade II, 11 patients) and neuropathy (≥ grade II, 5 patients). Toxicities were of sufficient clinical significance for use of a lower and well tolerated dose of 400 mg in currently accruing studies. © 2001 Cancer Research Campaignhttp://www.bjcancer.com


*British Journal of Cancer* (2001) **85**(7), 953–958
© 2001 Cancer Research Campaign
doi: 10.1054/ bjoc.2001.2025, available online at http://www.idealibrary.com on IDEAL

### The treatment of advanced renal cell cancer with high-dose oral thalidomide

**J Stebbing[1], C Benson[1], T Eisen[1], L Pyle[1], K Smalley[1], H Bridle[2], I Mak[3], F Sapunar[1], R Ahern[1] and ME Gore[1]**

[1]The Royal Marsden Hospital, Department of Medical Oncology, London SW3 6JJ; [2]North Middlesex Hospital, HEAL Research Department, London N18 1QX; [3]Chelsea and Westminster Hospital, Department of Clinical Neurophysiology, London SW10 9NH, UK

**Summary** Thalidomide is reported to suppress levels of several cytokines, angiogenic and growth factors including TNF-α, basic fibroblast growth factor (bFGF), vascular endothelial growth factor (VEGF) and interleukin-6 (IL-6). The resulting anti-angiogenic, immunomodulatory and growth suppressive effects form the rationale for investigating thalidomide in the treatment of malignancies. We have evaluated the use of high-dose oral thalidomide (600 mg daily) in patients with renal carcinoma. 25 patients (all men; median age, 51 years; range 34–76 years) with advanced measurable renal carcinoma, who had either progressed on or were not suitable for immunotherapy, received thalidomide in an escalating schedule up to a maximum dose of 600 mg daily. Treatment continued until disease progression or unacceptable toxicity were encountered. 22 patients were assessable for response. 2 patients showed partial responses (9%; 95% CI: 1–29), 7 (32%; 95% CI: 14–55) had stable disease for more than 6 months and a further 5 (23%; 95% CI: 8–45) had stable disease for between 3 and 6 months. We also measured levels of TNF-α, bFGF, VEGF, IL-6 and IL-12 before and during treatment. In patients with SD ≥ 3 months or an objective response, a statistically significant decrease in serum TNF-α levels was demonstrated ($P = 0.05$). The commonest toxicities were lethargy (≥ grade II, 10 patients), constipation (≥ grade II, 11 patients) and neuropathy (≥ grade II, 5 patients). Toxicities were of sufficient clinical significance for use of a lower and well tolerated dose of 400 mg in currently accruing studies. © 2001 Cancer Research Campaign http://www.bjcancer.com

**Keywords**: thalidomide; renal cell carcinoma

In 1971, Folkman showed that tumour growth was dependent on the formation of new blood vessels (Folkman, 1971), a process known as angiogenesis. Angiogenesis in carcinomas often correlates with the likelihood of the development of metastases and the prognosis of patients (Folkman, 1995). A number of different angiogenic and anti-angiogenic factors are known to be involved in the events that lead to endothelial cell proliferation, migration and invasion (Rak et al, 1996). These include TNF-α, bFGF, VEGF and various interleukins such as IL-6 and IL-12. It has recently been reported that the proteins prostate-specific antigen (Fortier et al, 1999), angiostatin (O'Reilly et al, 1997) and endostatin (O'Reilly et al, 1997) function as endogenous inhibitors of angiogenesis and can be produced in response to tumour proliferation. A corollary to the hypothesis that angiogenesis is central to tumour growth is that anti-angiogenic agents may represent a new strategy for controlling cancer growth.

Following extensive preclinical studies of angiogenesis inhibitors in animals, anti-angiogenic agents are now starting to be used in clinical practice (Folkman, 1995). Thalidomide has anti-angiogenic activity and can eradicate experimental tumours in mice (Ching et al, 1995). Its devastating teratogenic effects are probably in part due to the inhibition of blood vessel growth in the developing fetal limb bud (D'Amato et al, 1994). Recent publications suggest that its molecular effects are mediated through (i) oxidative DNA damage caused by the generation of hydroxyl-free radicals (Sauer et al, 2000), (ii) metabolites binding to guanine-cytosine rich growth factor promoter sites (Stephens and Filmore, 2000). (iii) the down-regulation of several cell-surface adhesion receptors (Thiel et al, 2000) and, (iv) enhancing the degradation of mRNA of peptide-signalling molecules such as TNF-α and FGF (Ching et al, 1995; Tavares et al, 1997; La Maestra et al, 2000).

Clinically, thalidomide has been used to treat such diverse conditions as graft-versus-host disease (Vogelsang et al, 1992), inflammatory bowel disease (Rhodes et al, 1997; Sands and Podolsky, 1999), refractory myeloma (Singhal et al, 1999) and AIDS-associated apthous ulcers (Jacobson et al, 1997), Kaposi's sarcoma (Mitsuyasu, 2000) and photodermatitis (Berger et al, 1995). The United States Food and Drug Administration approved the use of thalidomide in July 1998 for erythema nodosum leprosum, an inflammatory manifestation of leprosy (Sampaio et al, 1993). It may also have a role in treating cancer cachexia because high levels of TNF-α have previously been linked to cachexia and tumour-related malaise (Yoneda et al, 1991; Haslett, 1998).

A recent study (Eisen et al, 2000) at the Royal Marsden Hospital has evaluated the use of low dose (100 mg daily) thalidomide in 66 patients with melanoma, renal cell, ovarian or breast cancer. In patients with renal cell cancer, 3 responses were observed and a further 3 patients had stable disease for more than 3 months. There were no responses in melanoma, ovarian or breast cancer patients. Analysis of potential growth factors/angiogenic markers demonstrated a non-significant trend towards higher levels of VEGF being associated with disease progression. The occurrence of stable disease in patients with metastatic renal cancer is consistent with unpublished data from other groups. These results led to the phase II trial we now report which involves

*Received 14 March 2001*
*Revised 29 May 2001*
*Accepted 5 July 2001*

*Correspondence to:* ME Gore higher doses of oral thalidomide (600 mg daily) being given to patients with advanced renal cancer, with the concomitant evaluation of plasma angiogenic marker and growth factor concentrations.

### PATIENTS AND METHODS

### Patient identification and eligibility

Patients with histologically confirmed renal cell carcinoma were eligible for entry into this study. Eligibility criteria included the presence of metastatic disease that was measurable in at least one diameter greater than 1 cm by clinical examination or imaging. Patients were not allowed prior chemotherapy or immunotherapy within 4 weeks or major surgery within 3 weeks of entry into the study. Marker lesions were not allowed to have received radiotherapy at any time and patients with cerebral metastases were excluded. Patients had to be of ECOG/Zubrod performance status 0–2, older than 18 years and have a life expectancy greater than 3 months. No more than 3 prior treatment regimens, excluding adjuvant therapy were allowed.

Patients required adequate bone marrow reserve with white blood count greater than $3 \times 10^9$ $l^{-1}$, platelets greater than $100 \times 10^9$ $l^{-1}$, and a haemoglobin more than 10 g $dl^{-1}$. Patients were also required to have a normal serum creatinine or a glomerular filtration rate greater than 60 ml $min^{-1}$ as assessed by a $^{51}$Cr-EDTA clearance, in addition to adequate hepatic function defined as liver function tests less than twice the normal values, unless due to metastatic disease. Any woman of child-bearing potential had to have a negative pregnancy test prior to receiving thalidomide and use adequate contraception during the study. Patients with pre-existing peripheral neuropathy of WHO grade 2 or greater as assessed by sensory nerve action potential (SNAP) testing were not eligible (Fullerton and Kremer, 1961).

The protocol was approved by the Royal Marsden and North Middlesex Hospitals Ethics Committees and all patients gave written informed consent.

### Treatment

Patients received incremental doses of thalidomide up to a planned target dose of 600 mg daily (300 mg bd) according to the following schedule:

100 mg bd for 1 week
100 mg mane, 200 mg nocte for 1 week
200 mg bd for 2 weeks
200 mg mane, 300 mg nocte for 1 week
300 mg bd thereafter.

Thalidomide was supplied by Penn Pharmaceuticals Limited, Gwent, United Kingdom and treatment continued until there was evidence of disease progression, unacceptable toxicity, or the patient wished to stop treatment for any reason. Patients self-administered thalidomide more than 2 hours after food and a good fluid intake was advised.

Patients with unacceptable drowsiness or constipation had their thalidomide dose reduced by increments of 200 mg daily. Patients with SNAP tests that showed a reduction of ≥40% had their treatment stopped for 2 weeks and their dose of thalidomide reduced to 300 mg daily. A further SNAP test was performed 4–6 weeks after this dose modification and if the new test score was stable or improved, the dose of 300 mg was maintained. However, if the SNAP score decreased by ≥50% from baseline at any time, treatment was stopped.

### Assessment of response and toxicity

Baseline investigations included complete blood count, serum biochemistry, liver function tests, SNAP test, electrocardiogram and serum pregnancy test for women of child-bearing potential. A clinical examination, complete blood count and biochemistry were carried out every month while on treatment. The SNAP test was repeated at 1, 3, 6, 9 and 12 months during therapy and at 6 monthly intervals thereafter.

Patients underwent imaging or clinical measurement, as appropriate, to assess their disease status at the start of treatment and at 1, 3, 6, 9 and 12 months while on treatment. Response was measured according to standard International Union Against Cancer criteria. Toxicity was assessed according to WHO criteria at monthly intervals. Patients who received at least 4 weeks of treatment were assessable for response and all patients were assessable for toxicity. The response duration was defined as the time elapsed between the start of treatment with thalidomide and the date of disease progression or the last follow-up evaluation.

Plasma samples were taken in heparinised tubes at the start of treatment and stored at –70˚C. They were analysed using quantitative sandwich enzyme immunoassay kits for IL-6, IL-12, TNF-α, bFGF and VEGF levels (Quantikine kits, R&D systems, Abingdon, United Kingdom).

### Statistical analyses

A Gehan 2 stage design with 95% power at a 10% standard error was used. It was originally intended to recruit 9 patients and if there were no objective responses or stabilisation of disease the trial was to be terminated. There was a 5% probability that this would occur with a response or disease stabilising rate of 30%. Further recruitment of 16 patients was carried out in the second stage to obtain an estimate of the response rate with a standard error of 10%.

The non-parametric Wilcoxon test was employed to assess the significance of changes in plasma levels of angiogenic markers. The pre-treatment value of the angiogenic marker was compared with 6-month value (or the 3- or 9-month value if the 6-month value was unavailable). The one-month value was used as the pre-treatment value in the single patient in whom an immediate pre-treatment value was not obtained.

### RESULTS

### Patient characteristics

Between July 1998 and June 2000, 25 eligible patients with advanced renal cell cancer under the care of the Royal Marsden or the North Middlesex Hospital were entered into the study (median follow-up 20 months). The median age of the patients was 51 (range 34–76 years). Nearly all had undergone prior surgery and many had undergone extensive prior systemic treatment. The majority had a performance status of 1 and 7 patients had an ECOG performance status of 2 (Table 1). Progressive disease was identified in all patients within 16 weeks prior to study entry although this was not an inclusion criterion.

*© 2001 Cancer Research Campaign*

**Table 1** Baseline patient characteristics

| | Renal cancer (*n* = 25) |
|---|---|
| Male | 25 |
| Female | 0 |
| Age (range) | 51 (34–76 years) |
| ECOG performance status | |
| 0 | 3 |
| 1 | 15 |
| 2 | 7 |
| Previous treatment | |
| None | 1 |
| Surgery | 24 |
| Chemotherapy | 0 |
| Radiotherapy | 8 |
| Immunotherapy | 10 |
| Immuno-chemotherapy | 12 |
| Endocrine treatment | 5 |
| ≥2 of above treatments (excluding surgery) | 11 |
| Sites of metastasis | |
| Soft tissue/lymph node only | 4 |
| Lung ± soft tissue only | 8 |
| Viscera | 1 |
| Liver | 6 |
| Other (eg. spleen, adrenal, bone) | 7 |
| Number of disease/organ sites affected | |
| 1 | 4 |
| 2 | 15 |
| ≥3 | 6 |

### Tumour response

In this group, 2 patients achieved a PR and 12 had SD (SD ≥ 12 months in 2 patients, 6–12 months in 5 patients and 3–6 months in 5 patients, Table 2). 3 patients were not assessable for response due to early removal from the trial because of toxicity. The overall response rate by intention to treat was 8% (2/25) and for assessable patients the response rate was 9% (2/22). The median survival after commencing thalidomide in patients with renal cell cancer was 9 months and 4 patients currently remain on treatment.

The 2 patients with partial responses, one with small volume lung metastases, the other with retroperitoneal disease (nephrectomy bed recurrence and para-aortic lymph nodes) have maintained their partial responses for more than 12 months and both remain on a reduced dose of thalidomide (200 mg bd) due to toxicity.

We have updated results from our previous study using 100 mg daily as the median follow-up is now 36 months, as compared with a median follow-up of 20 months at publication (Eisen et al, 2000). In patients with advanced renal cell cancer, SD has been maintained for ≥ 12 an ≥ 6 months in 2 patients previously reported as having SD for 3–6 months. In both of these cases, treatment was stopped because 2 consecutive SNAP tests demonstrated worsening scores within the range of 40–50% (clinical grade II neuropathy). The combined median survival from the start of thalidomide therapy for all 40 assessable patients with renal cell cancer from both studies is 9 months (range 1–22+ months).

**Table 3** Toxicity (World Health Organisation Grade) in all 25 patients with advanced renal cancer, worst grade per patient

| | Grade I | Grade II | Grade III | Grade IV |
|---|---|---|---|---|
| Lethargy | 3 (12%) | 8 (32%) | 2 (8%) | – |
| Oedema | 2 (8%) | – | – | – |
| Constipation | – | 10 (40%) | 1 (4%) | – |
| Rash | 1 (5%) | – | – | – |
| Neuropathy | – | 4 (16%) | 1 (4%) | – |

### Toxicity

The most common side effects observed were lethargy, constipation and neuropathy (Table 3). Constipation generally responded well to standard measures although the lethargy observed was constant and more difficult to manage. No haematological toxicity was observed secondary to thalidomide.

6 patients with renal cell cancer did not reach 600 mg due to lethargy (grade II) at lower doses in 3 patients and death due to progressive disease in a further 3 patients who had an initial performance status of 2. Dose reductions were required in a further 11 patients with renal cancer who did reach the target dose. The toxicities leading to these reductions were as follows: 5 patients due to ≥ grade II neuropathy alone, 4 patients due to ≥ grade II lethargy alone, 1 patient due to constipation alone (grade III) and 1 patient due to a combination of constipation and lethargy (both grade II). One patient died suddenly after achieving SD for more than 6 months. The cause of death was established at post-mortem as being due to pulmonary emboli. 6 patients were in full time employment at the start of treatment and none had time off while on thalidomide.

### Angiogenic markers

Tables 4A and 4B illustrate the plasma concentrations of angiogenic markers, TNF-α and IL-6 in consecutive plasma samples (Table 4A, patients with SD ≥ 3 months or a PR; Table 4B, patients

**Table 2** Responses to treatment with thalidomide

| Tumour type | Assessable patients | PD | PR | SD ≥12 months | SD 6–12 months | SD 3–6 months | Median follow-up |
|---|---|---|---|---|---|---|---|
| Current study | | | | | | | |
| RCC | 22 | 5 (23%) | 2 (9%) | 2 (9%) | 5 (23%) | 5 (23%) | 20 months |
| 95% CI | | (8–45) | (1–29) | (1–29) | (8–45) | (8–45) | |
| Previous study | | | | | | | |
| RCC[24] | 18 | 12 (67%) (41–87) | 3 (17%) (4–41) | 0 | 0 | 3 (17%) (4–41) | 20 months |
| RCC[+] | 18* | 12 (67%) | 3 (17%) | 1 (6%) | 1 (6%) | 1 (6%) | 36 months |
| 95% CI | | (41–87%) | (4–41%) | (4–41%) | (4–41%) | (4–41%) | |

[+]Updated results of previous study (Eisen et al, 2000) *12 patients have died. 95% Confidence intervals are in given in brackets after percentages for each category. RCC = Renal Cell Cancer; PD = progressive disease; SD = stable disease; CI = Confidence interval.

© 2001 Cancer Research Campaign 

**Table 4A** Plasma concentrations of angiogenic markers, IL-6 and TNF-α in consecutive patients with renal cell carcinoma (SD ≥ 3 months or PR), before and during treatment with thalidomide. Values given are the mean of two individual measurements (pg $ml^{-1}$)

| | Time (response) | VEGF | bFGF | IL-6 | TNF-α |
|---|---|---|---|---|---|
| Patient 2 | 1 month (SD) | 196 | 32 | 130 | 23 |
| | 2 months (SD) | 297 | 2.5 | 5.5 | 5.4 |
| | 3 months (SD) | 127 | 8.5 | 7.9 | 9.6 |
| Patient 3 | pre-treatment | 486 | 63 | 2.7 | >32 |
| | 1 month (SD) | 93 | 2.4 | 22 | 3.3 |
| | 3 months (SD) | 169 | 8.8 | 3.2 | >32 |
| | 6 months (SD) | 97 | 3.6 | 20 | 2.1 |
| Patient 4 | pre-treatment | 361 | 12 | 161 | 4.4 |
| | 3 months (PR) | 334 | 16 | 71 | >32 |
| | 6 months (PR) | 275 | 20 | 124 | 3.6 |
| Patient 5* | pre-treatment | 171 | 3.4 | 1.8 | 5.7 |
| | 1 month (SD) | 160 | 5.2 | 2.1 | 7.7 |
| | 3 months (SD) | 140 | 15 | 2.6 | 6.2 |
| | 9 months (SD) | 146 | 2.5 | 4.6 | 3.9 |
| Patient 6 | pre-treatment | 79 | 5.5 | 1.5 | 3.8 |
| | 1 month (SD) | 80 | 8.4 | 1.4 | 4.3 |
| | 3 months (SD) | 590 | 47 | 7.6 | – |
| | 6 months (SD) | 72 | 5.5 | 1 | 4.1 |
| Patient 7 | pre-treatment | 197 | 61 | 5.3 | 2.8 |
| | 1 month (SD) | 317 | 42 | 15 | 20 |
| Patient 9 | pre-treatment | 58 | 4.9 | 13 | 2.7 |
| | 1 month (SD) | 161 | 22 | 5.9 | 3.8 |
| | 3 months (SD) | 98 | 5 | 5.8 | 2.3 |

*Stopped thalidomide after 7 months due to neurotoxicity.

**Table 4B** Plasma concentrations of angiogenic markers, IL-6 and TNF-α in consecutive patients with renal cell carcinoma (PD only), before and during treatment with thalidomide. Values given are the mean of two individual measurements (pg/$ml^{-1}$)

| | Time (Response) | VEGF | bFGF | IL-6 | TNF-α |
|---|---|---|---|---|---|
| Patient 1 | pre-treatment | 141 | 3 | 13 | 5.7 |
| | 2 months (SD) | 435 | 9.6 | 1 | 3.7 |
| | 3 months (PD) | 103 | 9.5 | 4.3 | 3.9 |
| Patient 8 | pre-treatment | 57 | 2.2 | 3.3 | 3.7 |
| | 1 month (PD) | 88 | 11 | 3 | 6.4 |
| Patient 10 | pre-treatment | 234 | 28 | 217 | 3.3 |
| | 1 month (SD) | 140 | 28 | 302 | >32 |
| | 3 months (PD) | 95 | 4.2 | 161 | >32 |

**Table 5** Ranges of plasma concentrations of angiogenic markers, IL-6 and TNF-α from patients with renal cell carcinoma, concentration ranges in pg $ml^{-1}$ and *P* values shown (Wilcoxon test)

| | VEGF | bFGF | IL-6 | TNF-α |
|---|---|---|---|---|
| PR/SD ≥3 months | | | | |
| Pre-treatment | 58–486 | 3.4–63 | 1.5–161 | 2.7– > 32 |
| During treatment | 72–590 | 2.4–47 | 1–130 | 2.1– > 32 |
| *P* value | 0.12 | 0.69 | 0.34 | 0.05 |
| All patients | | | | |
| Pre-treatment | 57–486 | 2.2–63 | 1.5–217 | 2.7– > 32 |
| During treatment | 72–590 | 2.4–47 | 1–302 | 2.1– > 32 |
| *P* value | 0.05 | 0.50 | 0.12 | 0.16 |

with PD). Patients with PD were often unavailable for repeated venepuncture and thus there were too few samples for meaningful statistical analysis from patients with renal cell cancer because so many of these patients progressed rapidly. The data illustrate the marked heterogeneity in levels of angiogenic markers, TNF-α and IL-6, in this small sample of 10 patients with renal cell cancer.

The non-parametric Wilcoxon test was used to analyse the results (Table 5). Levels of TNF-α demonstrated a statistically significantly decrease ($P = 0.05$) during thalidomide therapy in patients with SD ≥ 3 months or a PR. Plasma VEGF levels were notable for showing a statistically significant decrease during thalidomide therapy in all patients with renal cell cancer ($P = 0.05$). Plasma levels of IL-12 were undetectable (< 0.781 pg $ml^{-1}$) in all 10 patients.

Furthermore, the mean pre-treatment TFN-α level was 3.85 pg $ml^{-1}$ in 4 patients without clinical benefit from thalidomide (PR or SD ≥ 6 months) and 11.9 pg $ml^{-1}$ for 6 patients with clinical benefit, in whom cytokine levels were measured ($P = 0.06$, Mann–Whitney test).

### DISCUSSION

Thalidomide is reported to have particular anti-neoplastic activity in patients with renal cell carcinoma (Eisen et al, 2000), refractory myeloma (Singhal et al, 1999) and Kaposi's sarcoma (Mitsuyasu, 2000) although efficacy in other tumour types such as high-grade gliomas (Fine et al, 2000), breast or ovarian cancers and melanoma appears to be limited (Eisen et al, 2000). Results from this phase II trial of high-dose oral thalidomide confirm our previous experience, that this drug is active in previously treated patients with advanced renal cancer (Table 2) (Eisen et al, 2000).

In the 22 out of 25 patients with renal cell cancer who were assessable for response, 2 PRs were seen and SD ≥ 12 months was observed in 2 patients. The 2 patients who achieved PR remain in

 *© 2001 Cancer Research Campaign*

remission after more than 12 months and remain on 200 mg bd thalidomide which is well tolerated. A further 5 patients achieved stabilisation of their disease which lasted for more than 6 months. We consider disease stabilisation to be significant in this group as all patients had documented disease progression prior to study entry. By this criterion 9 patients (41%; 95% CI: 21–64) derived clinical benefit from this therapy.

The median survival of 9 months is the same as that observed in the updated results from our previous study. The combined data from our 2 studies in patients with advanced renal carcinoma ($n$ = 40) demonstrates a median survival of 9 months from the start of thalidomide treatment with 5 patients achieving a PR (23%) and a further 9 patients (41%) having stabilisation of their disease for ≥ 6 months. These results are comparable to those obtained in larger studies with single agent subcutaneously administered interferon-α in previously untreated patients (median survival 8.5 months, response rate 13%) (Medical Research Council Renal Cancer Collaborators, 1999).

A well-documented side-effect of thalidomide is peripheral neuropathy (Fullerton and Kremer, 1961) and dose reductions due to this were required in 5 patients. The most common side effects that we observed were constipation, which responded well to laxatives, neuropathy and lethargy. This last side effect was found to be constant and therefore more troublesome than the others. It should be noted that because of these side effects, 60% of our patients with renal cell cancer could not reach the planned target dose or required a dose reduction after a period of time on 600 mg daily. Patients subsequently tolerated a dose of 200 mg bd well with no significant (grade III or IV) toxicities.

One possible therapeutic target for thalidomide in renal cell carcinoma is TNF-α which is secreted by these tumours (Elsasser-Beile et al, 1999). This cytokine enhances neo-angiogenesis, augments the stimulation of renal cancer cells by IL-6 (Koo and Beldegrum, 1992; Konig et al, 1999), and is implicated in many of the non-specific systemic symptoms of advanced malignancy (Sampaio et al, 1993; Berger et al, 1995). In our patients who demonstrated a PR or SD ≥ 3 months, the associated decrease in plasma TNF-α with treatment was statistically significant ($P$ = 0.05). Furthermore, the mean pretreatment TNF-α plasma levels were 11.4 and 3.64 pg $ml^{-1}$ in those patients that did and did not obtain clinical benefit from treatment with thalidomide, respectively ($P$ = 0.06). These data are consistent with an anti-angiogenic action of thalidomide in this patient population but also suggest that thalidomide may be of particular use in the management of patients with renal cell cancer who secrete high levels of TNF-α.

Caution needs to be exercised when interpreting these findings because of the wide ranges in the plasma levels of angiogenic markers, TNF-α and IL-6 that we encountered in this small sample. Furthermore the results only just reached statistical significance and this could be explained by the high initial TNF-α plasma levels found in patients 2 and 3. A more accurate method of studying the biological effects of anti-angiogenic agents such as thalidomide involves the examination of biopsy material pre- and post-treatment to assess vascularity and the expression of angiogenic markers by in situ hybridisation techniques. Plasma levels of IL-6, IL-12, bFGF and VEGF did not significantly correlate with response to treatment in our study, a result observed in our previous trial which assayed clotted serum samples (Eisen et al, 2000).

The highest documented response rates in renal cell carcinoma are seen with a combination of interferon-α, 5-fluorouracil and interleukin-2 (IFI) (Lopez-Hanninen et al, 1996; Atzpodien et al, 1997). The IFI regimen, like other IL-2 based therapies, is associated with the induction of high levels of TNF-α (Weidmann et al, 1992). 12 of our patients had received this combination prior to study entry and a possible explanation for our results is that a proportion of patients demonstrated the documented phenomenon of a late response to biotherapy. This is unlikely as disease progression was observed in several patients who had IFI and objective disease stabilisation appeared to occur within 1 month of commencing thalidomide.

There are now increasing data suggesting synergy between anti-angiogenic agents and other cytotoxic drugs (Teicher et al, 1992, 1994, 1996). Furthermore, thalidomide has documented immunomodulatory effects and therefore it may prove valuable as part of a multi-modality anti-cancer strategy, rather than as a single therapy (Raje and Anderson, 1999; Fine et al, 2000). Future trials could also address its use as a single agent in the adjuvant setting. Our currently accuring studies in patients with advanced renal cell cancer use a target dose of 400 mg daily, largely because of the lethargy associated with 600 mg.

### REFERENCES

Atzpodien J, Kirchner H, Franzke A et al (1997) Results of a randomised clinical trial comparing SC interleukin-2, SC alpha-2A-interferon and iv bolus 5-fluorouracil against oral tamoxifen in progressive metastatic renal cell carcinoma patients. *Proc ASCO* **16** (abstr 1164): 326a

Berger TG, Hoffman C, Thierberg MD (1995) Prurigo nodularis and photosensitivity and AIDS: treatment with thalidomide. *J Am Acad Dermatol* **33**: 837–838

Ching LM, Xu ZF, Gummer BH et al (1995) Effect of thalidomide on tumour necrosis factor production and anti-tumour activity induced by 5,6-dimethylxantheone-4-acetic acid. *Br J Cancer* **72**: 339–343

D’Amato RJ, Loughnan MS, Flynn E and Folkman J (1994) Thalidomide is an inhibitor of angiogenesis. *Proc Natl Acad Sci USA* **91**: 4082–4085

Eisen T, Boshoff C, Mak I, Sapunar F, Vaughan MM, Pyle L, Johnston SRD, Ahern R, Smith IE and Gore ME (2000) Continuous low dose thalidomide: a phase II study in advanced melanoma, renal cell, ovarian and breast cancer. *Br J Cancer* **82**: 812–817

Elsasser-Beile U, Grussenmeyer T et al (1999) Semiquantitative analysis of Th1 and Th2 cytokine expression in CD3+, CD4+ and CD8+ renal-cell carcinoma-infiltrating lymphocytes. *Cancer Immunol Immunother* **48**: 204–208

Fine HA, Figg WD et al (2000) Phase II trial of the antiangiogenic agent thalidomide in patients with recurrent high-grade gliomas. *J Clin Oncol* **18**: 708–715

Folkman J (1971) Tumour angiogenesis: therapeutic implications. *N Engl J Med* **285**: 1182–1186

Folkman J (1995) Clinical applications of research on angiogenesis. *N Engl J Med* **333**: 1757–1763

Fortier AH, Nelson BJ, Grella DK and Holaday JW (1999) Antiangiogenic activity of prostate-specific antigen. *J Natl Cancer Inst* **91**: 1635–1640

Fullerton PM and Kremer M (1961) Neuropathy after intake of thalidomide (Distaval). *BMJ* **2**: 853–858

Haslett PA (1998) Anticytokine approaches to the treatment of anorexia and cachexia. *Semin Oncol* **25**: 53–57

Jacobson JM, Greenspan JS et al (1997) Thalidomide for the treatment of oral apthous ulcers in patients with human immunodeficiency virus infection. *N Engl J Med* **336**: 1487–1493

Koo, Beldegrum A (1992) Interleukin-6 and renal cell cancer: production, regulation and growth effects. *Cancer Immunol Immunother* **35**: 97–105

Konig B, Steinbach F et al (1999) The differential expression of proinflammatory cytokines IL-6, IL-8 and TNF-alpha in renal cell carcinoma. *Anticancer Res* **19**: 1519–1524

La Maestra L, Zaninoni A, Marriott JB et al (2000) The thalidomide analogue CC-3052 inhibits HIV-1 and tumour necrosis factor-alpha expression in acutely and chronically infected cells in vitro. *Clin Exp Immunol* **119**: 123–129

Lopez-Hanninen E, Kirchner H and Atzpodien J (1996) Interleukin-2 based home therapy of metastatic renal cell carcinoma: risks and benefits in 215 consecutive single institution patients. *J Urol* **155**: 19–25

Medical Research Council Renal Cancer Collaborators (1999) Interferon-α and survival in metastatic renal carcinoma: early results of a randomised controlled trial. *Lancet* **353**: 14–17

© 2001 Cancer Research Campaign

Mitsuyasu RT (2000) Update on the pathogenesis and treatment of Kaposi's sarcoma. *Curr Opin Oncol* **12**: 174–180

O'Reilly MS, Holmgren L, Shing Y et al (1994) Angiostatin: a novel angiogenesis inhibitor that mediates the suppression of metastases by a Lewis Lung carcinoma. *Cell* **79**: 315–328

O'Reilly MS, Boehm T, Shing Y et al (1997) Endostatin: an endogenous inhibitor of angiogenesis and tumour growth. *Cell* **88**: 277–285

Raje N and Anderson K (1999) Thalidomide – a revival story. *N Engl J Med* **341**: 1606–1608

Rak J, Filmus J and Kerbel RS (1996) Reciprocal paracrine interactions between tumour cells and endothelial cells: the angiogenic progression' hypothesis. *Eur J Cancer* **32A**: 2438–2450

Rhodes J, Thomas G and Evans BK (1997) Inflammatory bowel disease management. Some thoughts on future drug developments. *Drugs* **53**: 189–194

Sampaio EP, Kaplan G et al (1993) The influence of thalidomide on the clinical and immunologic manifestation of erythema nodosum leprosum. *J Infect Dis* **168**: 408–414

Sands BE and Podolsky DK (1999) New life in a sleeper: thalidomide and Crohn's disease. *Gastroenterology* **117**: 1485–1488

Sauer H, Gunther J et al (2000) Thalidomide inhibits angiogenesis in embryoid bodies by the generation of hydroxyl radicals. *Am J Pathol* **156**: 151

Singhal S et al (1999) Antitumour activity of thalidomide in refractory multiple myeloma. *N Engl J Med* **341**: 1565–1571

Stephens TD and Filmore BJ (2000) Hypothesis: thalidomide embryopathy-proposed mechanism of action. *Teratology* **61**: 189–195

Tavares JL, Wangoo A, Dilworth P et al (1997) Thalidomide reduces tumour necrosis factor-alpha production by human alveolar macrophages. *Respir Med* **91**: 31–39

Teicher BA, Sotomayor EA, Huang ZD et al (1992) Antiangiogenic agents potentiate cytotoxic cancer therapies against primary and metastatic disease. *Cancer Res* **52**: 6702–6704

Teicher BA, Holder SA, Ara G et al (1994) Potentiation of cytotoxic cancer therapies by TNP-40 alone and with other anti-angiogenic agents. *Cancer* **57**: 1–6

Teicher BA, Holder SA, Ara G et al (1996) Comparison of several antiangiogenic regimens alone and with cytotoxic therapies in the Lewis lung carcinoma. *Cancer Chemother Pharmacol* **38**: 169–177

Thiel R, Kastner U and Neubert R (2000) Expression of adhesion receptors on rat limb bud cells and results of treatment with a thalidomide derivative. *Life Sci* **66**: 133–141

Vogelsang GB, Farmer ER, Hess AD et al (1992) Thalidomide for the treatment of chronic graft-versus-host disease. *N Engl J Med* **326**: 1055–1058

Weidmann E, Bergmann L, Stock J et al (1992) Rapid cytokine release in cancer patients treated with interleukin-2. *J Immunother* **12**: 123–131

Yoneda T et al (1991) Evidence that tumour necrosis factor plays a pathogenetic role in the paraneoplastic syndromes of cachexia, hypercalcemia, and leukocytosis in a human tumour nude mice. *J Clin Invest* **87**: 977–985

 *© 2001 Cancer Research Campaign*

## References

[PDF_00499] Berger T. G., Hoffman C., Thieberg M. D. (1995). Prurigo nodularis and photosensitivity in AIDS: treatment with thalidomide.. J Am Acad Dermatol.

[PDF_00501] Ching L. M., Xu Z. F., Gummer B. H., Palmer B. D., Joseph W. R., Baguley B. C. (1995). Effect of thalidomide on tumour necrosis factor production and anti-tumour activity induced by 5,6-dimethylxanthenone-4-acetic acid.. Br J Cancer.

[PDF_00504] D'Amato R. J., Loughnan M. S., Flynn E., Folkman J. (1994). Thalidomide is an inhibitor of angiogenesis.. Proc Natl Acad Sci U S A.

[PDF_00506] Eisen T., Boshoff C., Mak I., Sapunar F., Vaughan M. M., Pyle L., Johnston S. R., Ahern R., Smith I. E., Gore M. E. (2000). Continuous low dose Thalidomide: a phase II study in advanced melanoma, renal cell, ovarian and breast cancer.. Br J Cancer.

[PDF_00510] Elsässer-Beile U., Grussenmeyer T., Gierschner D., Schmoll B., Schultze-Seemann W., Wetterauer U., Schulte Mönting J. (1999). Semiquantitative analysis of Th1 and Th2 cytokine expression in CD3+, CD4+, and CD8+ renal-cell-carcinoma-infiltrating lymphocytes.. Cancer Immunol Immunother.

[PDF_00521] FULLERTON P. M., KREMER M. (1961). Neuropathy after intake of thalidomide (distaval).. Br Med J.

[PDF_00513] Fine H. A., Figg W. D., Jaeckle K., Wen P. Y., Kyritsis A. P., Loeffler J. S., Levin V. A., Black P. M., Kaplan R., Pluda J. M. (2000). Phase II trial of the antiangiogenic agent thalidomide in patients with recurrent high-grade gliomas.. J Clin Oncol.

[PDF_00517] Folkman J. (1995). Seminars in Medicine of the Beth Israel Hospital, Boston. Clinical applications of research on angiogenesis.. N Engl J Med.

[PDF_00515] Folkman J. (1971). Tumor angiogenesis: therapeutic implications.. N Engl J Med.

[PDF_00519] Fortier A. H., Nelson B. J., Grella D. K., Holaday J. W. (1999). Antiangiogenic activity of prostate-specific antigen.. J Natl Cancer Inst.

[PDF_00523] Haslett P. A. (1998). Anticytokine approaches to the treatment of anorexia and cachexia.. Semin Oncol.

[PDF_00525] Jacobson J. M., Greenspan J. S., Spritzler J., Ketter N., Fahey J. L., Jackson J. B., Fox L., Chernoff M., Wu A. W., MacPhail L. A. (1997). Thalidomide for the treatment of oral aphthous ulcers in patients with human immunodeficiency virus infection. National Institute of Allergy and Infectious Diseases AIDS Clinical Trials Group.. N Engl J Med.

[PDF_00528] Koo A. S., Armstrong C., Bochner B., Shimabukuro T., Tso C. L., deKernion J. B., Belldegrum A. (1992). Interleukin-6 and renal cell cancer: production, regulation, and growth effects.. Cancer Immunol Immunother.

[PDF_00530] König B., Steinbach F., Janocha B., Drynda A., Stumm M., Philipp C., Allhoff E. P., König W. (1999). The differential expression of proinflammatory cytokines IL-6, IL-8 and TNF-alpha in renal cell carcinoma.. Anticancer Res.

[PDF_00533] La Maestra L., Zaninoni A., Marriott J. B., Lazzarin A., Dalgleish A. G., Barcellini W. (2000). The thalidomide analogue CC-3052 inhibits HIV-1 and tumour necrosis factor-alpha (TNF-alpha) expression in acutely and chronically infected cells in vitro.. Clin Exp Immunol.

[PDF_00536] Lopez Hänninen E., Kirchner H., Atzpodien J. (1996). Interleukin-2 based home therapy of metastatic renal cell carcinoma: risks and benefits in 215 consecutive single institution patients.. J Urol.

[PDF_00545] Mitsuyasu R. T. (2000). Update on the pathogenesis and treatment of Kaposi sarcoma.. Curr Opin Oncol.

[PDF_00550] O'Reilly M. S., Boehm T., Shing Y., Fukai N., Vasios G., Lane W. S., Flynn E., Birkhead J. R., Olsen B. R., Folkman J. (1997). Endostatin: an endogenous inhibitor of angiogenesis and tumor growth.. Cell.

[PDF_00547] O'Reilly M. S., Holmgren L., Shing Y., Chen C., Rosenthal R. A., Moses M., Lane W. S., Cao Y., Sage E. H., Folkman J. (1994). Angiostatin: a novel angiogenesis inhibitor that mediates the suppression of metastases by a Lewis lung carcinoma.. Cell.

[PDF_00552] Raje N., Anderson K. (1999). Thalidomide--a revival story.. N Engl J Med.

[PDF_00554] Rak J., Filmus J., Kerbel R. S. (1996). Reciprocal paracrine interactions between tumour cells and endothelial cells: the 'angiogenesis progression' hypothesis.. Eur J Cancer.

[PDF_00557] Rhodes J., Thomas G., Evans B. K. (1997). Inflammatory bowel disease management. Some thoughts on future drug developments.. Drugs.

[PDF_00559] Sampaio E. P., Kaplan G., Miranda A., Nery J. A., Miguel C. P., Viana S. M., Sarno E. N. (1993). The influence of thalidomide on the clinical and immunologic manifestation of erythema nodosum leprosum.. J Infect Dis.

[PDF_00562] Sands B. E., Podolsky D. K. (1999). New life in a sleeper: thalidomide and Crohn's disease.. Gastroenterology.

[PDF_00564] Sauer H., Günther J., Hescheler J., Wartenberg M. (2000). Thalidomide inhibits angiogenesis in embryoid bodies by the generation of hydroxyl radicals.. Am J Pathol.

[PDF_00566] Singhal S., Mehta J., Desikan R., Ayers D., Roberson P., Eddlemon P., Munshi N., Anaissie E., Wilson C., Dhodapkar M. (1999). Antitumor activity of thalidomide in refractory multiple myeloma.. N Engl J Med.

[PDF_00568] Stephens T. D., Fillmore B. J. (2000). Hypothesis: thalidomide embryopathy-proposed mechanism of action.. Teratology.

[PDF_00570] Tavares J. L., Wangoo A., Dilworth P., Marshall B., Kotecha S., Shaw R. J. (1997). Thalidomide reduces tumour necrosis factor-alpha production by human alveolar macrophages.. Respir Med.

[PDF_00578] Teicher B. A., Holden S. A., Ara G., Korbut T., Menon K. (1996). Comparison of several antiangiogenic regimens alone and with cytotoxic therapies in the Lewis lung carcinoma.. Cancer Chemother Pharmacol.

[PDF_00572] Teicher B. A., Sotomayor E. A., Huang Z. D. (1992). Antiangiogenic agents potentiate cytotoxic cancer therapies against primary and metastatic disease.. Cancer Res.

[PDF_00581] Thiel R., Kastner U., Neubert R. (2000). Expression of adhesion receptors on rat limb bud cells and results of treatment with a thalidomide derivative.. Life Sci.

[PDF_00584] Vogelsang G. B., Farmer E. R., Hess A. D., Altamonte V., Beschorner W. E., Jabs D. A., Corio R. L., Levin L. S., Colvin O. M., Wingard J. R. (1992). Thalidomide for the treatment of chronic graft-versus-host disease.. N Engl J Med.

[PDF_00586] Weidmann E., Bergmann L., Stock J., Kirsten R., Mitrou P. S. (1992). Rapid cytokine release in cancer patients treated with interleukin-2.. J Immunother (1991).

[PDF_00588] Yoneda T., Alsina M. A., Chavez J. B., Bonewald L., Nishimura R., Mundy G. R. (1991). Evidence that tumor necrosis factor plays a pathogenetic role in the paraneoplastic syndromes of cachexia, hypercalcemia, and leukocytosis in a human tumor in nude mice.. J Clin Invest.

